# Preprocedural Serum Albumin and Early Contrast-Associated Acute Kidney Injury After Elective Carotid Artery Stenting: Incremental Prognostic Information Beyond the PCI-Derived Mehran Score

**DOI:** 10.3390/diagnostics16213441

**Published:** 2026-10-22

**Authors:** Gökhan Karataş, Ezgi Kula, Burcu Orug Köseer

**Affiliations:** 1 Department of Radiology, Trakya University, Edirne 22030, Turkey; 2 Department of Neurology, Trakya University, Edirne 22030, Turkey

**Keywords:** carotid artery stenting, serum albumin, Mehran score, estimated glomerular filtration rate, acute kidney injury, risk stratification

## Abstract

**Background/Objectives:** We assessed whether preprocedural serum albumin adds prognostic information to the PCI-derived Mehran score for early contrast-associated acute kidney injury (CA-AKI) after elective carotid artery stenting (CAS). **Methods:** This single-centre retrospective cohort included 121 consecutive elective patients (January 2020–April 2026). The primary model combined the reconstructed Mehran score with albumin < 3.5 g/dL, a conventional threshold fixed in advance. Because the score uses actual contrast volume, the combined model applies only after CAS. The outcome used KDIGO serum creatinine thresholds (≥0.3 mg/dL or ≥1.5 times baseline) from routine second-day testing. Internal validation used 1000 bootstrap resamples. **Results:** Early CA-AKI occurred in 32 patients (26.4%). Adjusted odds ratios were 1.35 per Mehran point (95% CI 1.13–1.60) and 11.68 for albumin < 3.5 g/dL (95% CI 4.14–32.97). AUC increased from 0.768 to 0.865 (95% CI 0.794–0.937; likelihood ratio χ^2^ = 24.58, *p* < 0.001). Corrected AUC, calibration slope, and Brier score were 0.860, 0.96, and 0.132. In exploratory decision curves (20% threshold), corrected net benefit was 0.168 for the combined model and 0.133 for Mehran alone. **Conclusions:** Low preprocedural albumin added prognostic information to the PCI-derived score and may help select patients for closer renal surveillance after CAS. External validation and prospective impact studies are needed before clinical use.

## 1. Introduction

Postprocedural deterioration in kidney function is a clinically relevant complication after angiographic and endovascular procedures. Contemporary terminology distinguishes contrast-associated acute kidney injury (CA-AKI) from contrast-induced acute kidney injury (CI-AKI), because an increase in serum creatinine after contrast exposure establishes a temporal association but does not, by itself, establish causality. In studies without an appropriate non-exposed control group, CA-AKI is therefore the more appropriate descriptive term [1].

The Mehran score was developed and validated to estimate the risk of contrast-related kidney injury after percutaneous coronary intervention (PCI). Its original eight components comprise hypotension, intra-aortic balloon pump use, congestive heart failure, age > 75 years, anaemia, diabetes mellitus, contrast volume, and renal function [2]. Because carotid artery stenting (CAS) differs from PCI in its procedural and haemodynamic context, the transportability of the original Mehran score to CAS remains uncertain [3,4]. Here, we use it as a PCI-derived comparator in a CAS cohort. It is not an established CAS prediction tool, and this study does not validate its original risk estimates.

Evidence regarding acute kidney injury after CAS remains limited. In patients with chronic kidney disease undergoing CAS, AKI occurred in approximately one in five patients and was independently associated with periprocedural haemodynamic depression, whereas contrast volume was similar in patients with and without AKI [3]. A larger prospective registry of patients undergoing proximal-protected CAS also reported a substantial incidence of AKI and identified procedural blood pressure changes among its independent predictors [5]. A review has described kidney injury after CAS as an underestimated, multifactorial complication [6]. A CAS-specific study also reported an association between the neutrophil-to-lymphocyte ratio and contrast-induced acute kidney injury after carotid artery stenting [7]. Most CAS studies have examined individual clinical or procedural risk factors, not whether a preprocedural biomarker adds prognostic information to a PCI-derived composite score [3,5,7].

Serum albumin may reflect several dimensions of systemic and physiological vulnerability, including nutritional and inflammatory status, endothelial integrity, and effective circulating volume [8,9]. Lower preprocedural serum albumin has been associated with contrast-associated kidney injury in coronary intervention cohorts, and a meta-analysis of eight studies including 18,687 patients found hypoalbuminaemia to be associated with a higher risk of CA-AKI [8,10]. More recently, lower preprocedural albumin and higher hsCRP were independently associated with CA-AKI in patients undergoing coronary angiography or PCI [11]. Albumin was routinely measured before elective CAS at our institution, making it an accessible candidate marker. However, whether preprocedural serum albumin provides prognostic information beyond the PCI-derived Mehran score in patients undergoing CAS has not been adequately evaluated.

The primary objective was to estimate the association between preprocedural serum albumin and early creatinine-defined CA-AKI after adjustment for the reconstructed PCI-derived Mehran score. Discrimination, calibration, and net benefit were examined as exploratory model updating. Because the score includes the actual contrast volume, the combined model can be applied only after CAS, before the scheduled postprocedural creatinine measurement. It is not a fully preprocedural prediction tool.

## 2. Materials and Methods

### 2.1. Study Design and Population

This retrospective cohort study was conducted in the Department of Interventional Radiology, Trakya University Faculty of Medicine. All patients who underwent carotid artery stenting (CAS) between January 2020 and April 2026 were identified from departmental procedure logs, and consecutive case ascertainment was verified against the hospital information system and PACS records. All procedures included in the analytical cohort were performed electively during planned admissions; no patient underwent stenting during the acute phase of ischaemic stroke. Accordingly, “symptomatic disease” referred to ipsilateral neurological symptoms occurring before referral for elective CAS rather than to intervention performed in the acute setting.

The study was approved by the Clinical Research Ethics Committee (approval number TÜTF-GOBAEK 2026-448), which waived the requirement for individual informed consent because of the retrospective design.

Patients were included if CAS was performed electively during a planned admission within the study period and if a preprocedural serum albumin value, baseline and routine second-day creatinine values, and all variables needed to reconstruct the Mehran score were available. Urgent CAS and CAS during the acute phase of ischaemic stroke were outside the target population, and records lacking predictor or outcome data were ineligible. Of 122 identified patients, one urgent external referral was excluded because the clinical and laboratory records were unavailable after transfer on the procedure day, leaving 121 elective cases. Active infection, malignancy, and chronic liver disease were not systematically adjudicated and were not used as exclusion criteria. Analyses used complete cases without imputation; missing procedure duration affected only the sensitivity analysis that used it. Reporting followed STROBE and the TRIPOD items relevant to regression estimation and internal validation.

### 2.2. Procedure and Periprocedural Protocol

All procedures were performed by interventional radiologists via a transfemoral approach under local anaesthesia. A distal embolic-protection filter was used in all patients. Predilatation and postdilatation were performed when clinically indicated, and self-expanding carotid stents were deployed across the target lesion. During the study period, Omnipaque, Ultravist, and Xenetix were used; all were non-ionic, low-osmolar iodinated contrast agents with an iodine concentration of 300 mg/mL. Contrast agent selection depended on stock availability and was not recorded at the individual patient level.

According to the institutional protocol, dual antiplatelet therapy with acetylsalicylic acid and clopidogrel was initiated at least one week before CAS. Intravenous unfractionated heparin was administered as a 5000-IU bolus at the start of the procedure, with additional 1000-IU doses given hourly when required under activated clotting time monitoring. Isotonic saline was prescribed at approximately 1 mL/kg/h for 6–12 h before and 6–12 h after the procedure; the duration could be shortened at the discretion of the treating physician in patients considered at risk of volume overload. The actual volume infused was not recorded and therefore could not be analysed as a study variable.

Brief bradycardia or hypotension related to balloon manipulation was treated promptly. Sustained hypotension was assessed against the original Mehran definition [2]. The severity and duration of transient balloon-related bradycardia or hypotension were not systematically recorded.

### 2.3. Laboratory Variables, eGFR, and Mehran Score

Baseline serum creatinine, albumin, haematocrit, haemoglobin, LDH, CRP, and differential leukocyte counts were measured before CAS during the scheduled admission. Albumin therefore preceded the intervention, but the exact time from blood sampling to CAS could not be recovered reliably for every patient. We could not confirm same-day or within-24-h sampling and did not impute an interval. Active infection, malignancy, and chronic liver disease were not systematically adjudicated and could not be used as adjustment variables. Diabetes mellitus was defined by a documented physician diagnosis or antidiabetic treatment, anaemia by preprocedural haematocrit using the original Mehran criteria, and congestive heart failure by documented clinical diagnosis [2].

All laboratory measurements were performed in the same laboratory. Serum creatinine, albumin, LDH and CRP were measured on a Roche Cobas C701 analyser (Mannheim, Germany) with the manufacturer’s kits. eGFR was recalculated from baseline serum creatinine, age, and sex using the 2009 CKD-EPI creatinine equation without applying the race coefficient [12]. The 2021 CKD-EPI creatinine equation was used in a sensitivity analysis [13].

The PCI-derived Mehran score was reconstructed using the original components and weighting scheme: 5 points each for qualifying hypotension, intra-aortic balloon pump use, and congestive heart failure; 4 points for age > 75 years; 3 points for anaemia; 3 points for diabetes mellitus; 1 point for each complete 100-mL increment in contrast volume; and 2, 4, or 6 points for eGFR values of 40–59, 20–39, or <20 mL/min/1.73 m^2^, respectively [2]. The contrast component used the total volume actually given during each procedure, so it was known only after CAS. We kept the original component weights, estimated the intercept and slope of the Mehran-only logistic model in this cohort, and did not use the published PCI category risks as CAS probabilities.

Hypoalbuminaemia was defined as serum albumin < 3.5 g/dL (35 g/L), a conventional clinical definition used in earlier prognostic research [14]. The threshold was chosen on clinical grounds before the outcome associations were examined and was kept fixed in all primary model analyses and bootstrap resamples. It was not selected by ROC optimisation or by maximising statistical significance, and it is not a CAS-specific optimal cut-off. Continuous linear and spline models assessed the association without a binary threshold. Only the preprocedural concentration was studied; within-patient changes in albumin were not evaluated.

### 2.4. Outcome Definition

Serum creatinine was remeasured on the morning of the second day after CAS under the institutional protocol, nominally about 48 h after the procedure; exact sampling times were not verified for every patient. Early CA-AKI was defined with the KDIGO serum-creatinine thresholds: an increase of ≥0.3 mg/dL or a value ≥ 1.5 times baseline at that measurement [1,15]. Either threshold was sufficient. Comparisons used the reported two-decimal creatinine values in decimal arithmetic, without further rounding, so that values exactly at a diagnostic boundary were classified correctly.

Serial urine output was not available, so the urine output criterion could not be applied. The endpoint is therefore a single early creatinine-based measure, not complete serial KDIGO ascertainment including urine output. As a sensitivity analysis, we also applied the historical definition of contrast-induced nephropathy, an increase in serum creatinine of ≥25% or ≥0.5 mg/dL from baseline [2].

### 2.5. Statistical Analysis

Continuous variables were presented as medians and interquartile ranges, and categorical variables as numbers and percentages. Between-group comparisons were performed using the Mann–Whitney U test for continuous variables and the chi-square or Fisher’s exact test for categorical variables, as appropriate. Univariable associations were evaluated using logistic regression and are reported as odds ratios (ORs) with 95% confidence intervals (CIs).

The sample comprised all consecutive eligible cases from January 2020 to April 2026; there was no a priori power calculation and no target number of events or non-events. The 32 patients with and 89 without CA-AKI are outcome-defined groups within one cohort, not separately sampled cases and controls, and no balancing, oversampling, or undersampling was performed. The primary logistic model had two predictor parameters (Mehran score and albumin < 3.5 g/dL), or 16 events per parameter excluding the intercept. We report this ratio descriptively; it does not show that the sample size was adequate. Predictors and the clinical threshold were fixed in advance, without outcome-driven variable selection. Continuous linear and spline models were secondary or exploratory, and split-sample validation was not used because the sample was small [16].

Incremental information was tested with a likelihood ratio test comparing the nested Mehran-only and combined models; this, rather than an AUC-comparison *p* value, was the main inferential comparison [17,18]. AUCs and their descriptive 95% confidence intervals were calculated with the nonparametric DeLong method. The AUC difference is reported with a paired percentile-bootstrap interval, refitting both models in each resample. Adding the Mehran score to the albumin-only model was an exploratory reverse comparison.

Internal validation used 1000 ordinary patient-level bootstrap resamples of 121 patients (random seed 20261012). In each resample, all regression coefficients were refitted with the specified predictors and the fixed albumin threshold, and the refitted model was evaluated both in its bootstrap sample and in the original cohort. The mean difference between the two (the optimism) was subtracted from the apparent AUC and Brier score. Calibration-in-the-large was estimated with the linear predictor as an offset, and the calibration slope by logistic recalibration. Because their apparent values are 0 and 1, the corrected estimates are the mean recalibration estimates of the resampled models in the original cohort. The percentile range of these calibration slopes describes internal uncertainty. All five models used the same resamples and procedure [19].

The exploratory restricted cubic spline had three knots at the 10th, 50th, and 90th albumin percentiles (3.3, 3.9, and 4.5 g/dL), giving two albumin degrees of freedom (one linear, one non-linear) and, with the Mehran score, three predictor parameters. The number and placement of knots were set for this revision without reference to the outcome association, and the knot values were recalculated in each bootstrap sample. Non-linearity was tested against the linear albumin model (one degree of freedom). Predicted probabilities were plotted at the median Mehran score (6) above a histogram of albumin values, so no odds ratio reference concentration was needed. Sixteen patients had albumin ≤ 3.3 g/dL and 14 had albumin ≥ 4.5 g/dL. Sensitivity analyses used Firth bias-reduced logistic regression, the historical outcome definition, CKD-EPI 2021, and separate adjustment for CRP, calendar year, or procedure duration. CRP was analysed on its original scale and as ln(CRP + 1), which allowed the six recorded zero values to be included.

An exploratory component-based model entered albumin < 3.5 g/dL with age, continuous eGFR, anaemia, diabetes mellitus, and contrast volume. It was also fitted with Firth penalised regression and assessed by bootstrap resampling. This six-parameter model had about 5.3 events per predictor parameter and was treated as an exploratory sensitivity analysis; neither Firth regression nor bootstrapping removes the need for a larger independent cohort. Non-convergent or unstable bootstrap fits were counted and reported. A separate contrast-separated model included albumin < 3.5 g/dL, the Mehran score without its contrast component, and actual contrast volume as a continuous covariate.

Exploratory decision curve analysis compared the refitted Mehran-only, albumin-only, and combined models for a hypothetical decision to add renal surveillance after CAS [20,21]. Net benefit was TP/N − (FP/N) × pt/(1 − pt), where pt is the risk threshold, and model curves were corrected for optimism at each threshold with the same bootstrap procedure. The 5% to 50% threshold range was chosen during revision as an illustrative range; these are not validated treatment or surveillance thresholds. The reference strategies were additional surveillance for all patients and no model-triggered additional surveillance, both assuming that routine renal care continues. No formal risk-reclassification categories or clinical impact outcomes were analysed.

Performance of albumin < 3.5 g/dL was summarised using sensitivity, specificity, and predictive values with Wilson 95% confidence intervals. The albumin–CRP association was assessed using Spearman correlation. Revision analyses used Python 3.12.14, NumPy 2.3.5, SciPy 1.17.0, scikit-learn 1.8.0, and Matplotlib 3.10.8. Tests were two-sided, with *p* < 0.05 as the nominal significance threshold; exploratory analyses were not adjusted for multiple comparisons. Firth estimates used the Jeffreys-penalised likelihood with Wald confidence intervals. All analyses added during revision are labelled exploratory.

## 3. Results

### 3.1. Cohort Characteristics

Of 122 consecutive CAS patients, one urgently treated external referral patient was excluded because transfer on the procedure day precluded access to the required clinical and laboratory data. The analytical cohort comprised 121 elective patients; 97 (80.2%) were men, and median age was 71 years (IQR 64–75). Median eGFR was 79.5 mL/min/1.73 m^2^ (IQR 63.9–90.5), serum albumin was 3.9 g/dL (IQR 3.4–4.2), and the Mehran score was 6 (IQR 4–8; range 0–13). Albumin was <3.5 g/dL in 35 patients (28.9%). No patient had documented qualifying hypotension, intra-aortic balloon pump use, or congestive heart failure. Variables used in the primary model and outcome classification were complete; procedure duration was available for 117 patients (96.7%). Group-specific characteristics are shown in Table 1.

### 3.2. Outcome Incidence and Baseline Comparisons

Creatinine-defined early CA-AKI occurred in 32 of 121 patients (26.4%). Twenty-four met only the absolute ≥ 0.3 mg/dL criterion and eight met both the absolute and the ≥1.5-fold criteria; no patient met only the relative criterion. At the scheduled measurement, 29 patients met creatinine-based stage 1 thresholds, two met stage 2, and one met stage 3. The largest absolute increase was 2.74 mg/dL. These stages rely on a single creatinine measurement, without urine output or renal replacement therapy data. Absolute and relative creatinine changes are given in Table 1.

Patients with early CA-AKI had higher age, Charlson Comorbidity Index, Mehran score, CRP, and LDH values and lower eGFR, haemoglobin, and albumin values than patients without early CA-AKI (Table 1). Early CA-AKI occurred in 22/35 patients (62.9%) with albumin < 3.5 g/dL and 10/86 (11.6%) with albumin ≥ 3.5 g/dL. The Spearman correlation between albumin and CRP was −0.31 (*p* < 0.001).

At the conventional threshold, albumin < 3.5 g/dL identified 22 of the 32 patients who developed early CA-AKI (sensitivity 68.8%, 95% CI 51.4–82.0) and was absent in 76 of the 89 patients who did not (specificity 85.4%, 95% CI 76.6–91.3). The positive and negative predictive values were 62.9% (95% CI 46.3–76.8) and 88.4% (95% CI 79.9–93.6), respectively.

### 3.3. Univariable Associations with Early CA-AKI

Univariable odds ratios were 1.38 per Mehran point (95% CI 1.18–1.60), 0.10 per 1 g/dL higher albumin (95% CI 0.03–0.31), and 12.86 for albumin <3.5 g/dL (95% CI 4.97–33.30; all *p* < 0.001). Results for all evaluated predictors are shown in Table 2.

### 3.4. Primary Combined Model, Discrimination, and Calibration

In the primary combined model, the OR was 1.35 per Mehran point (95% CI 1.13–1.60; *p* < 0.001) and 11.68 for albumin <3.5 g/dL (95% CI 4.14–32.97; *p* < 0.001).

**Figure 1:**
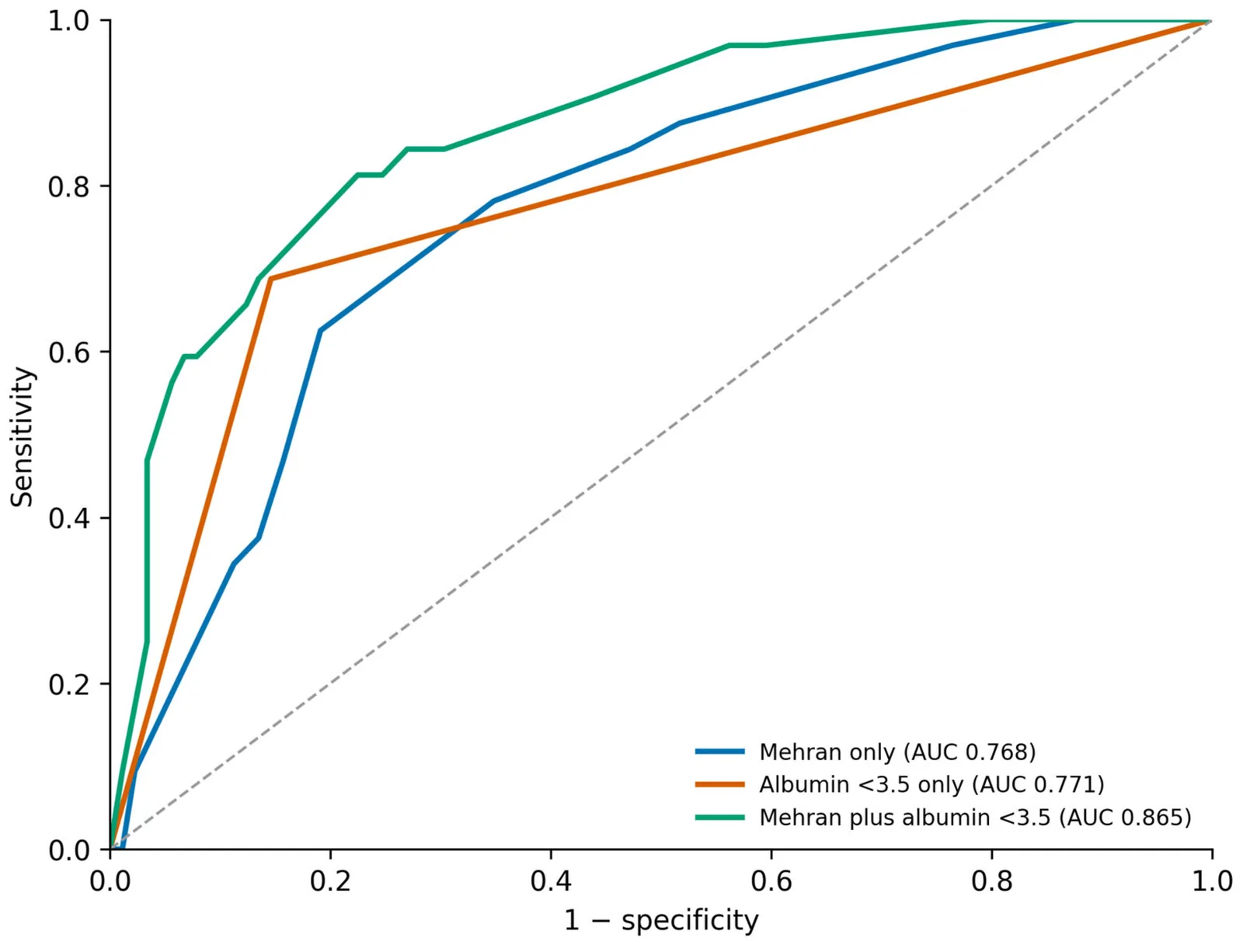
Receiver operating characteristic curves for creatinine-defined early CA-AKI. Curves compare the refitted Mehran-only logistic model, preprocedural albumin < 3.5 g/dL alone, and the combined model. Apparent AUCs were 0.768, 0.771, and 0.865, respectively; 95% intervals and corrected estimates are given in Table 3. All curves refer to the same 121 patients.

The AUC was 0.768 (0.677–0.859) for the Mehran-only model, 0.771 (0.681–0.860) for albumin < 3.5 g/dL, and 0.775 (0.668–0.883) for continuous albumin alone. The primary combined AUC was 0.865 (0.794–0.937), with a corrected AUC of 0.860 (Figure 1). The likelihood ratio statistic was χ^2^ = 24.58 (*p* < 0.001); ΔAUC was 0.097 (paired bootstrap 95% interval 0.033–0.190). Adding the Mehran score to the binary albumin-only model increased AUC by 0.095.

**Table 3:** Discrimination and prediction error of the primary and exploratory models.

Model and Predictor Specification	AUC (95% CI)	Corrected AUC	Brier Score	Corrected Brier Score
Model 1: Mehran score	0.768 (0.677–0.859)	0.768	0.164	0.170
Model 2: Albumin < 3.5 g/dL	0.771 (0.681–0.860)	0.770	0.141	0.147
Model 3, primary: Mehran + albumin < 3.5 g/dL	0.865 (0.794–0.937)	0.860	0.122	0.132
Model 4: Mehran + continuous linear albumin	0.824 (0.728–0.920)	0.812	0.135	0.145
Exploratory: Mehran + spline albumin	0.865 (0.793–0.937)	0.845	0.127	0.139

All models used 121 patients and 32 events. AUC intervals are descriptive nonparametric DeLong 95% confidence intervals for the apparent predictions. Corrected AUC and Brier scores come from 1000 bootstrap resamples with coefficient refitting; a small negative optimism estimate can arise from sampling variability. Excluding intercepts, Models 3 and 4 have two predictor parameters and the spline model has three. Association estimates are given in Section 3.3 and Section 3.4 and Table 2. The clinical cut-off was not re-selected during validation. AUC, area under the curve; CI, confidence interval.

**Figure 2:**
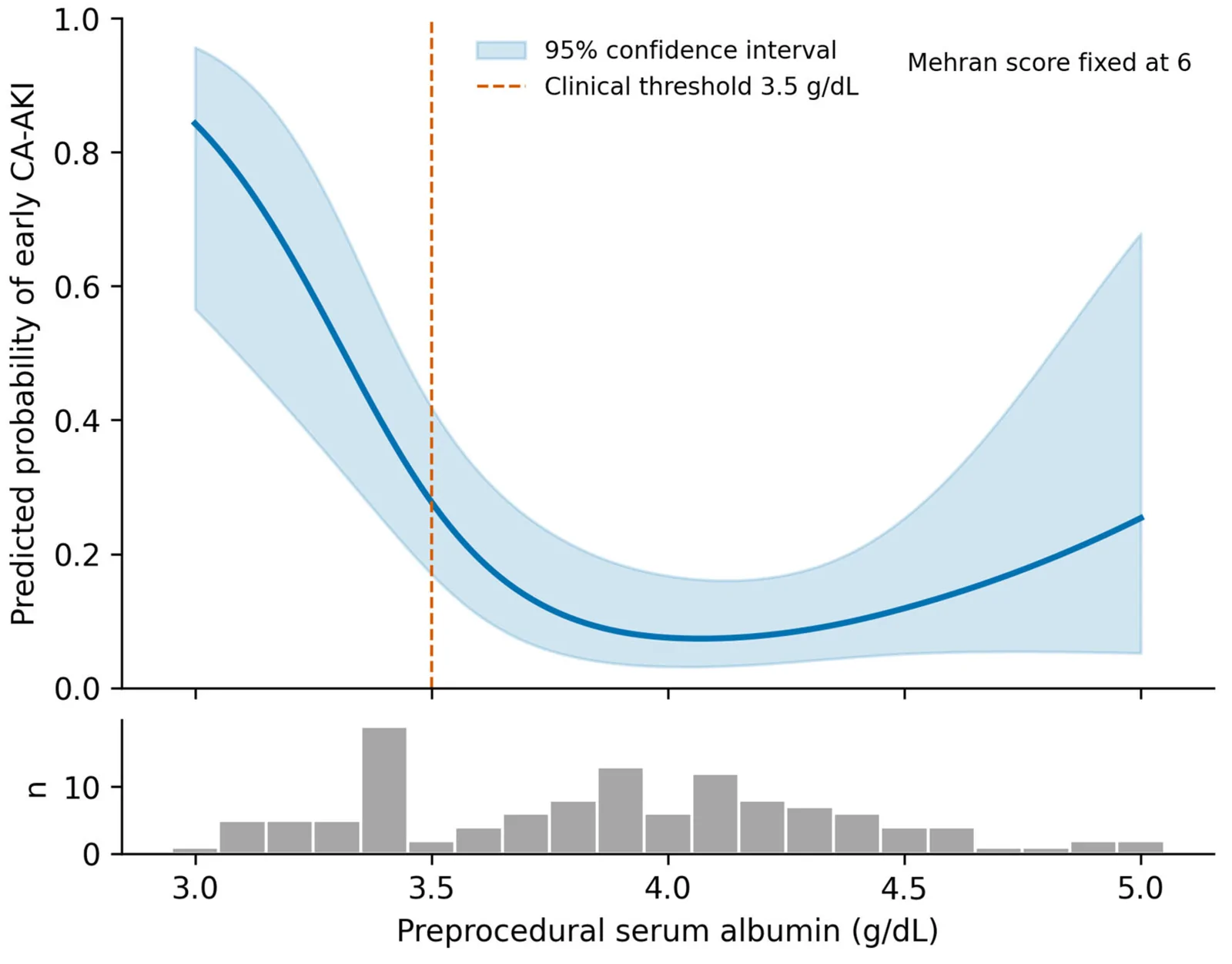
Exploratory non-linear albumin association with creatinine-defined early CA-AKI. A restricted cubic spline with three knots at 3.3, 3.9, and 4.5 g/dL is shown at Mehran score 6. Shading denotes pointwise 95% confidence intervals. The lower panel shows the observed albumin distribution; the dashed line marks the conventional 3.5 g/dL threshold, which was not estimated from these data.

In the linear continuous model, the adjusted OR was 1.31 (1.12–1.54) per Mehran point and 0.14 (0.04–0.46) per 1 g/dL higher albumin. The spline model had AUC 0.865 (0.793–0.937), corrected AUC 0.845, and likelihood ratio χ^2^ = 8.96 for its non-linear term (*p* = 0.003; Figure 2).

**Figure 3:**
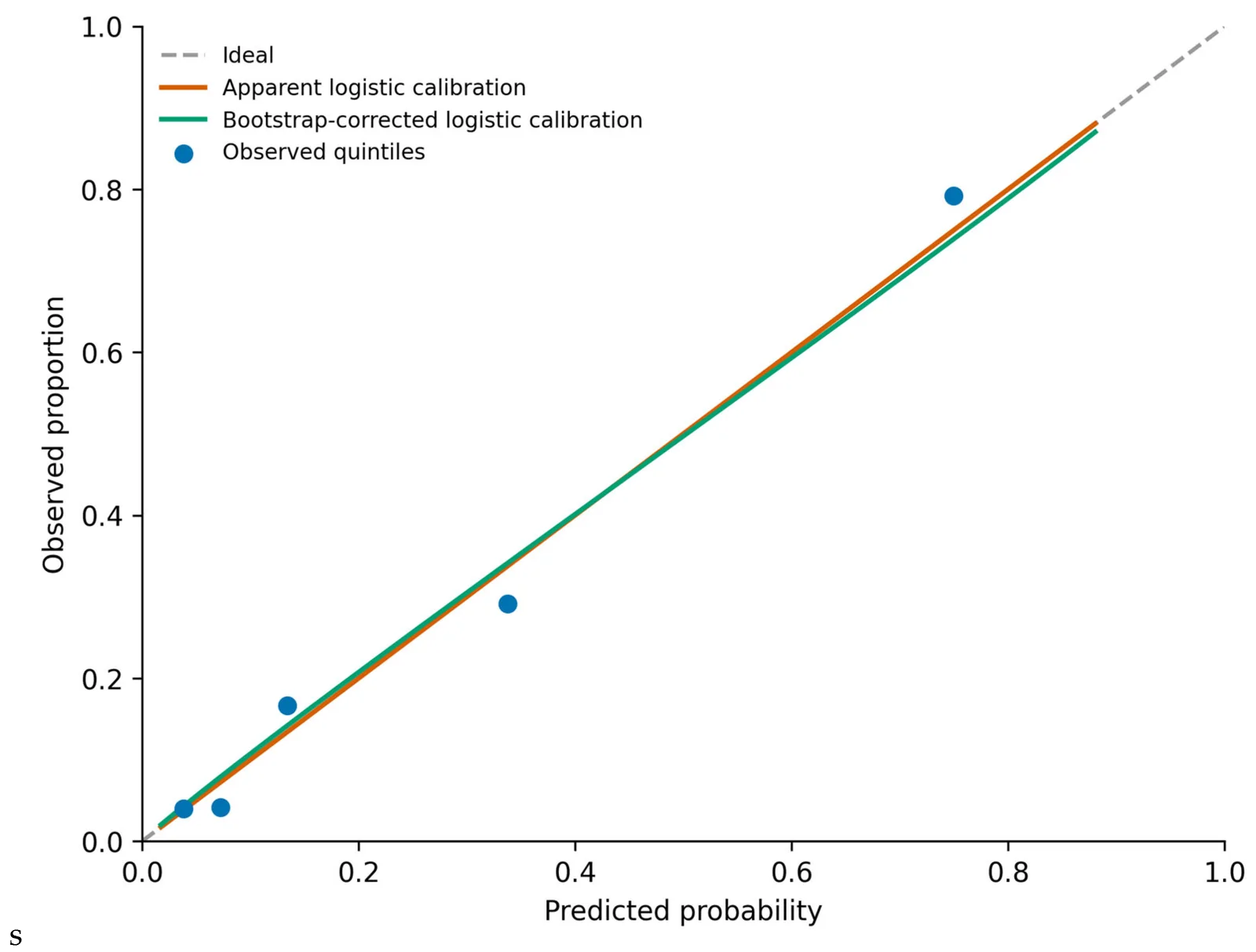
Internal calibration of the combined model. The plot shows apparent joint logistic recalibration, bootstrap-averaged joint logistic recalibration, the ideal line, and observed event proportions in five equally sized groups of ordered predicted probabilities. Apparent logistic calibration lies on the ideal line by construction for the fitted maximum-likelihood model. The corrected slope was 0.96, but its wide percentile interval (0.64–1.37) shows limited precision. Calibration-in-the-large is reported in the text.

The primary apparent Brier score was 0.122, with a corrected Brier score 0.132. The corrected calibration slope was 0.96, with a bootstrap test 2.5th–97.5th percentile interval of 0.64–1.37; the corrected calibration intercept in the large was 0.01. All 1000 resamples produced valid estimates for each of the five models in Table 3. Logistic calibration curves and observed quintiles are shown in Figure 3.

For reproducibility, the primary model equation in original units was logit(p) = −4.036559 + 0.297807 × Mehran score + 2.458216 × I(albumin < 3.5 g/dL), with p = 1/[1 + exp(−logit(p))]. The equation reproduces the apparent fitted probabilities. We provide it so that others can test it, not as a validated clinical calculator.

### 3.5. Sensitivity Analyses

Table 4 summarises the sensitivity analyses. Across the Firth, historical outcome, CKD-EPI 2021, and CRP-, calendar-year-, and procedure-duration-adjusted models, albumin ORs ranged from 9.68 to 13.42. The historical definition (≥25% or ≥0.5 mg/dL) identified 33 events and agreed with the primary definition in 120 of 121 patients; decimal comparison kept one patient whose creatinine rose from 1.32 to 1.65 mg/dL, an exact 25% increase. With CKD-EPI 2021, the Mehran renal function category changed in eight patients.

In the contrast-separated model, with contrast volume entered as a continuous variable, ORs were 1.34 (95% CI 1.13–1.60) for the Mehran score without its contrast component, 10.89 (95% CI 3.73–31.81) for albumin < 3.5 g/dL, and 1.05 per 10 mL of contrast (95% CI 0.92–1.21; *p* = 0.444).

In the component-based exploratory model, the adjusted albumin OR was 11.95 (3.82–37.41), or 9.64 (3.25–28.56) with Firth regression. Of 1000 bootstrap resamples, 994 gave stable convergent estimates and six were unstable or non-convergent. Among the successful fits, the median albumin OR was 14.33, with a 2.5th–97.5th percentile range of 4.35–98.03. With ln(CRP + 1) adjustment, the albumin OR was 9.43 (3.18–27.95), and transformed CRP had *p* = 0.247.

### 3.6. Exploratory Descriptive Risk Distribution

Observed early CA-AKI frequencies by Mehran category and albumin status are shown in Table 5. No patient had a Mehran score ≥ 16.

### 3.7. Exploratory Decision Curve Analysis

**Figure 4:**
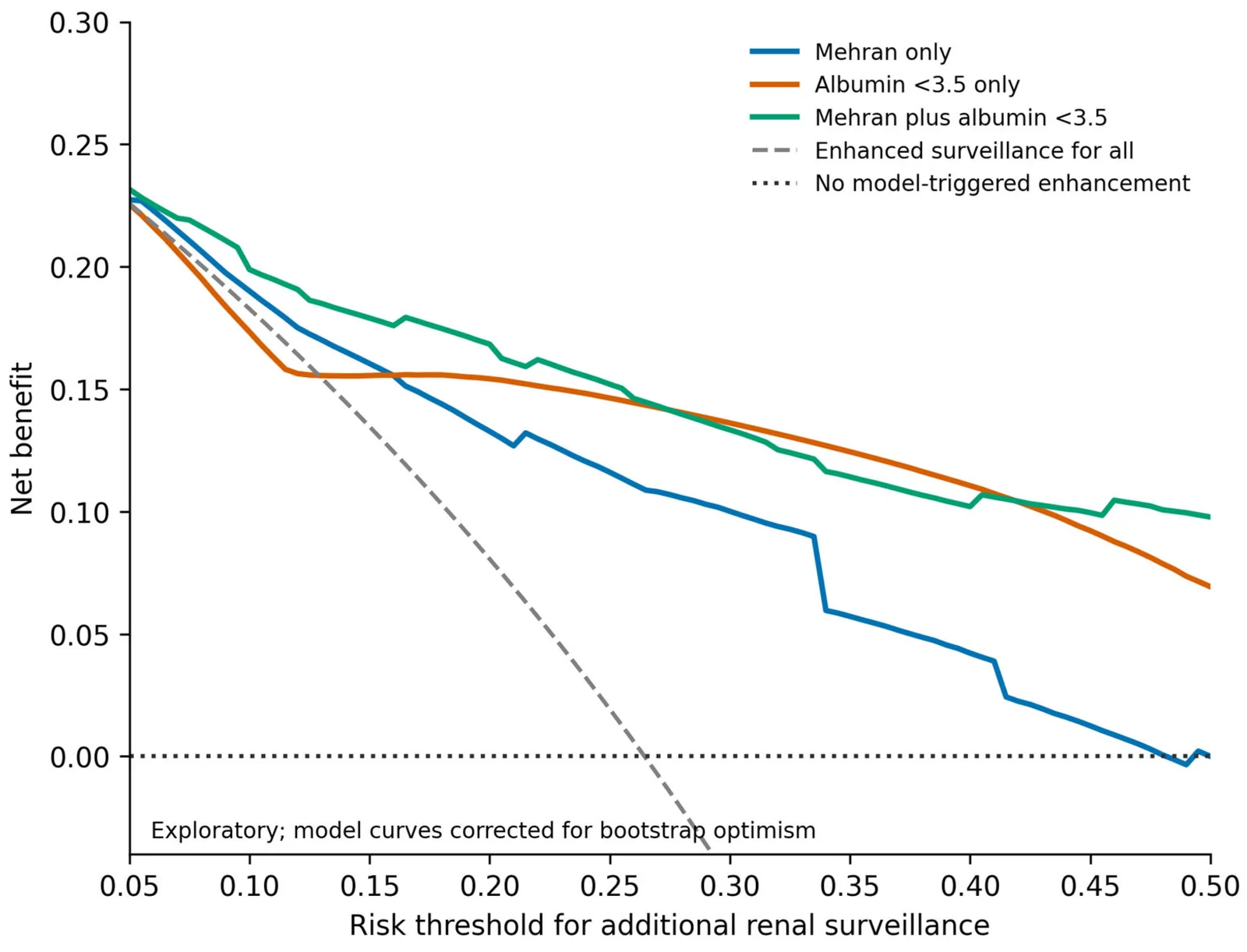
Exploratory decision curves for additional renal surveillance after CAS. Model curves show net benefit corrected for optimism by 1000 bootstrap resamples, with coefficients re-estimated in each resample. The 5–50% risk threshold range is illustrative. “Additional surveillance for all” and “no model-triggered enhancement” are hypothetical additions to routine care; routine creatinine testing continues in both. The curves do not show improved patient outcomes or define a surveillance threshold.

At risk thresholds of 10%, 20%, and 30%, corrected net benefits were 0.199, 0.168, and 0.133 for the combined model; 0.190, 0.133, and 0.100 for the Mehran-only model; and 0.173, 0.154, and 0.136 for the albumin-only model, respectively. At 40%, the combined and albumin-only values were 0.102 and 0.111. Figure 4 shows the model curves and the two reference strategies. Additional surveillance for all patients had net benefits of 0.183, 0.081, and −0.051 at 10%, 20%, and 30%.

## 4. Discussion

Preprocedural hypoalbuminaemia was associated with early creatinine-defined CA-AKI after adjustment for the reconstructed PCI-derived Mehran score, and adding albumin improved apparent discrimination. The wide confidence interval of the OR limits conclusions about effect size. This is a prognostic association study with exploratory model updating; we did not develop a validated CAS-specific score, and the analysis does not show that the original PCI risk estimates apply to CAS.

Early CA-AKI occurred in 26.4% of our elective cohort. Donahue et al. studied 126 patients with chronic kidney disease and reported AKI in 26 (21%), defined as a creatinine increase of ≥0.3 mg/dL at 48 h; haemodynamic depression and the Mehran risk score were independent predictors [3]. Their cohort was restricted to CKD, whereas only 22 of our patients had baseline eGFR < 60 mL/min/1.73 m^2^. Their finding supports the relevance of the score in CAS but does not show that the original PCI probabilities are calibrated for CAS.

Pucciarelli et al. reported AKI in 155 of 456 patients (34%) after proximal-protected CAS, using early creatinine thresholds similar to ours [5]. A post-procedural systolic pressure drop >50 mmHg, diabetes, and dyslipidaemia were independent predictors; the contrast volume-to-creatinine clearance ratio was higher in patients with AKI but was not an independent predictor. Their mean baseline eGFR was 76.7 mL/min/1.73 m^2^, so a high early biochemical event rate can occur in cohorts not restricted to CKD. The different embolic protection (distal filters in our cohort) and our incomplete haemodynamic data limit mechanistic comparison.

Ösken et al. reported a lower incidence, with 26 events among 389 CAS patients (6.6%), and found NLR to be independently associated with kidney injury [7]. In our cohort, NLR did not differ significantly between groups, whereas hypoalbuminaemia remained associated with the outcome after score adjustment. Patient selection, measurement timing, renal and inflammatory profiles, and chance may explain the difference. Diabetes and anaemia were common in our patients, and routine testing mostly detected stage 1 creatinine changes. These comparisons do not show that the observed rate reflects persistent kidney injury or that albumin outperforms NLR in other CAS populations.

Because the combined model uses actual contrast exposure, it can be completed only after CAS. We propose it for research on early postprocedural assessment, before routine second-day creatinine testing, for example, to prioritise additional surveillance. Albumin itself is known before the intervention; the combined model is not. We kept the original Mehran weights and fitted only the logistic intercept and slope locally, so no new integer-point scale was created. A preprocedural PCI model such as Mehran-2 was not assessed in this cohort [22].

An association between serum albumin and CA-AKI has been reported in other contrast-exposed populations. A meta-analysis of eight observational studies with 18,687 patients linked hypoalbuminaemia to a higher CA-AKI risk, while noting substantial heterogeneity and that observational data cannot establish causality [10]. In patients undergoing coronary angiography or PCI, lower preprocedural albumin and higher hsCRP were independently associated with CA-AKI [11]. These results cannot be transferred directly to CAS, but they are consistent with the association we observed.

The direction of the association agrees with earlier studies. The larger estimate in our cohort may reflect differences in case mix, outcome definition, adjustment, and chance, and its size cannot be judged reliably against a pooled estimate from heterogeneous non-CAS populations [10]. The OR is not an elevenfold causal effect and not a risk ratio; low albumin may capture unmeasured systemic illness and physiological vulnerability.

Low albumin may act as a marker of the patient’s clinical state before CAS. Nutritional status, inflammation, and volume status may link low albumin to susceptibility to kidney injury [10,11]. Proposed pathways include impaired effective renal perfusion and reduced antioxidant capacity [9,23], although these pathways were not measured here. The association persisted after CRP adjustment, so measured CRP did not fully explain it; this does not show a causal effect of albumin itself.

The continuous analyses suggest a non-linear association, but the spline is exploratory. The 3.5 g/dL threshold was chosen on clinical grounds before outcome analysis and was not optimised for CAS. It is easy to interpret but discards information within each category and does not imply an abrupt biological threshold. The upturn in risk at high albumin should not be interpreted biologically: few patients had high values, and the corrected spline AUC was lower than the apparent AUC.

The contrast volume estimate was compatible with both little association and a clinically relevant increase in risk. This fits a multifactorial picture in which haemodynamic changes also contribute to kidney injury after CAS [3,4,5]. Transient bradycardia or hypotension from carotid sinus stimulation may contribute even when sustained hypotension qualifying for Mehran points is absent. Our data cannot separate the effects of contrast exposure, haemodynamic disturbance, and baseline systemic vulnerability.

The corrected calibration slope was close to one, but its percentile interval was wide. The grouped calibration points and fitted curves describe this cohort only; apparent logistic calibration is ideal by construction and says nothing about calibration elsewhere. Bootstrap correction estimates internal optimism and is not a substitute for independent calibration.

One possible use, which needs prospective evaluation, is to identify patients who may need renal surveillance beyond the routine second-day creatinine test, such as an additional creatinine check, a review of nephrotoxic drugs and haemodynamic events, and earlier renal follow-up when clinically indicated. The decision curves illustrate the benefit–burden trade-off for this hypothetical decision. The combined model was not better than albumin alone at every threshold, so any added benefit depends on the threshold. All patients received routine hydration and scheduled testing, and no management strategies were compared. The findings therefore do not justify automatic changes in hydration, albumin infusion, cancelling CAS, or less routine monitoring in patients with a low predicted risk. External validation and prospective assessment of management impact are needed.

### 4.1. Strengths

This study adds CAS-specific evidence on whether a routine laboratory test contributes information beyond a PCI-derived composite score. Consecutive case ascertainment, protocol-based creatinine measurement, and use of the actual contrast volume give the analysis a consistent basis. Discrimination and calibration were assessed together, with bootstrap internal validation and alternative model specifications. Because all predictors are collected routinely, and the full model equation, AUC intervals, and decision curves are reported, the combined model can be tested in independent CAS cohorts and in studies of renal surveillance.

### 4.2. Limitations

This retrospective single-centre cohort had 32 events. Although the primary model had only two predictor parameters and no variable selection, its estimates are imprecise. The spline and the six-parameter component model are more prone to overfitting; six component-model bootstrap fits were unstable or non-convergent, and the OR distribution of the successful fits was wide. Firth regression and bootstrap validation do not remove these limitations. Dichotomising albumin discards information, and neither external validation nor clinical impact has been assessed.

The outcome rested on one scheduled second-day creatinine value, and exact sampling times were not verified for every patient. Albumin and baseline creatinine were drawn before CAS during the same planned admission, but the interval to the procedure was incompletely documented. This adds uncertainty to exposure measurement and may cause outcome misclassification. Urine output, recovery before discharge, dialysis, length of stay, and longer-term renal outcomes were not systematically available, so the reported stages describe creatinine thresholds at one early measurement rather than full clinical AKI severity.

Actual hydration volume and the severity and duration of transient haemodynamic events were not recorded. Clinicians could shorten hydration in patients they judged at risk of volume overload, and this judgement may relate to the same vulnerability that low albumin reflects, leaving possible confounding by indication. Haemodynamic depression is especially relevant after CAS and remains a central unmeasured pathway here. Medication exposure, proteinuria, active infection, malignancy, and chronic liver disease were not systematically adjudicated, heart failure relied on recorded diagnoses, and the contrast agent used in each patient was not recorded. These gaps leave room for residual confounding and misclassification.

The comparator was developed for PCI, and three of its components were absent in this elective CAS cohort. Fitting the score model locally does not show that the original PCI probabilities transfer to CAS. Incomplete ejection-fraction and glucose data prevented assessment of Mehran-2. Neither the conventional albumin threshold nor the illustrative decision-curve thresholds have been validated for CAS decisions, and the net-benefit curves are internal estimates that do not represent observed treatment effects and are uncertain in this small sample. The findings cannot be extended to urgent or haemodynamically unstable CAS.

## 5. Conclusions

In this elective CAS cohort, preprocedural albumin <3.5 g/dL was associated with early creatinine-defined CA-AKI and added prognostic information to the reconstructed PCI-derived Mehran score. The combined model can be applied after the procedure and may help identify patients who could benefit from additional renal surveillance. Its decision-related benefit was exploratory and depended on the threshold. External validation and prospective impact studies are needed before the threshold or the model is used to guide clinical management.

## Figures and Tables

**Table 1:** Clinical, laboratory, and procedural characteristics according to early contrast-associated acute kidney injury status.

Variable	No CA-AKI (*n* = 89)	CA-AKI (*n* = 32)	*p* Value
Age, years	69 (62–72)	76 (71–80)	<0.001
Age > 75 years	14 (15.7)	16 (50.0)	<0.001
Male sex	72 (80.9)	25 (78.1)	0.736
Symptomatic disease	75 (84.3)	30 (93.8)	0.232
Hypertension	62 (69.7)	26 (81.2)	0.207
Diabetes mellitus	64 (71.9)	26 (81.2)	0.299
Hyperlipidaemia	32 (36.0)	11 (34.4)	0.873
Coronary artery disease	29 (32.6)	10 (31.2)	0.890
Current or former smoker	51 (57.3)	17 (53.1)	0.683
Charlson Comorbidity Index	4 (3–5)	5 (4–6)	0.003
Baseline serum creatinine, mg/dL	0.91 (0.76–1.05)	1.00 (0.83–1.13)	0.060
CKD-EPI 2009 eGFR, mL/min/1.73 m^2^	82.5 (67.5–93.7)	71.0 (57.6–84.3)	0.004
eGFR < 60 mL/min/1.73 m^2^	13 (14.6)	9 (28.1)	0.089
Haemoglobin, g/dL	13.0 (11.6–13.9)	12.1 (11.1–12.9)	0.016
Mehran-defined anaemia	47 (52.8)	26 (81.2)	0.005
Serum albumin, g/dL	4.0 (3.7–4.3)	3.4 (3.3–3.8)	<0.001
Albumin < 3.5 g/dL	13 (14.6)	22 (68.8)	<0.001
C-reactive protein, mg/L	1.9 (0.6–6.9)	4.8 (1.6–14.8)	0.014
Lactate dehydrogenase, U/L	199 (171–231)	230 (201–303)	0.003
Neutrophil-to-lymphocyte ratio	2.50 (1.87–3.41)	2.71 (2.07–4.01)	0.290
Carotid stenosis, %	95 (80–99)	95 (75–99)	0.940
Contrast volume, mL	117 (94–140)	123 (100–156)	0.105
Contrast volume > 150 mL	16 (18.0)	9 (28.1)	0.224
Procedure duration, min ^a^	44 (36–55)	49 (36–58)	0.805
Mehran score	5 (4–7)	8 (7–11)	<0.001
Mehran score ≥ 11	10 (11.2)	11 (34.4)	0.003
Absolute change in serum creatinine, mg/dL ^b^	0.03 (−0.02 to 0.10)	0.39 (0.33–0.46)	—
Relative change in serum creatinine, % ^b^	3.8 (−2.2 to 11.0)	39.1 (33.6–48.9)	—

Data are presented as median (interquartile range) for continuous variables and n (%) for categorical variables. Continuous variables were compared using the Mann–Whitney U test. Categorical variables were compared using the chi-square test or Fisher’s exact test, as appropriate. ^a^ Procedure duration was available for 117 patients; all other variables had complete data. ^b^ Absolute and relative creatinine changes were calculated from baseline and postprocedural measurements. No group-comparison *p* values are reported for these outcome-derived changes. Abbreviations: CA-AKI, contrast-associated acute kidney injury; CKD-EPI, Chronic Kidney Disease Epidemiology Collaboration; eGFR, estimated glomerular filtration rate.

**Table 2:** Univariable logistic regression analyses for early contrast-associated acute kidney injury.

Predictor	OR	95% CI	*p* Value
Mehran score, per 1-point increase	1.38	1.18–1.60	<0.001
Albumin, per 1 g/dL increase	0.10	0.03–0.31	<0.001
Albumin < 3.5 g/dL	12.86	4.97–33.30	<0.001
Age, per 10-year increase	2.92	1.60–5.35	<0.001
CKD-EPI 2009 eGFR, per 10 mL/min/1.73 m^2^ increase	0.72	0.56–0.92	0.009
Mehran-defined anaemia	3.87	1.45–10.32	0.007
Diabetes mellitus	1.69	0.62–4.61	0.303
Charlson Comorbidity Index, per 1-point increase	1.38	1.08–1.77	0.011
C-reactive protein, per 1 mg/L increase	1.03	1.00–1.07	0.054
Contrast volume, per 10 mL increase	1.09	0.98–1.22	0.111

Each predictor was evaluated in a separate univariable logistic regression model with early CA-AKI as the dependent variable. Odds ratios for continuous variables correspond to the increments specified in the table. All *p* values are two-sided. Abbreviations: CA-AKI, contrast-associated acute kidney injury; CI, confidence interval; CKD-EPI, Chronic Kidney Disease Epidemiology Collaboration; eGFR, estimated glomerular filtration rate; OR, odds ratio.

**Table 4:** Sensitivity and exploratory analyses of the primary model.

Analysis	*n*	Score OR (95% CI)	Albumin < 3.5 g/dL OR (95% CI)	AUC (95% CI)	Additional Result
Primary model (reference)	121	1.35 (1.13–1.60)	11.68 (4.14–32.97)	0.865 (0.794–0.937)	—
Firth penalised logistic regression	121	1.33 (1.12–1.57)	10.68 (3.86–29.54)	0.865 (0.794–0.937)	—
Historical CIN definition	121	1.33 (1.12–1.58)	13.42 (4.74–38.04)	0.871 (0.801–0.941)	33 events; agreement with primary definition in 120/121 patients
CKD-EPI 2021 eGFR used for Mehran scoring	121	1.34 (1.13–1.60)	11.74 (4.17–33.03)	0.866 (0.795–0.937)	Renal function category changed in 8 patients
Additional adjustment for CRP	121	1.36 (1.14–1.61)	9.68 (3.27–28.65)	0.867 (0.796–0.938)	CRP *p* = 0.332
Additional adjustment for calendar year	121	1.34 (1.13–1.60)	11.83 (4.17–33.56)	0.865 (0.794–0.936)	Calendar year *p* = 0.744
Additional adjustment for procedure duration	117 ^c^	1.34 (1.13–1.60)	10.38 (3.64–29.60)	0.857 (0.783–0.931)	Procedure duration *p* = 0.573
Contrast-separated model ^a^	121	1.34 (1.13–1.60)	10.89 (3.73–31.81)	0.862 (0.787–0.936)	Contrast volume OR 1.05 per 10 mL (0.92–1.21); *p* = 0.444
Exploratory component-based model ^b^	121	^b^	11.95 (3.82–37.41)	0.873 (0.800–0.946)	Firth albumin OR 9.64 (3.25–28.56); successful bootstrap fits 994/1000; median OR 14.33 (percentile range 4.35–98.03)

Each row represents a separate analysis. Unless otherwise specified, models included the reconstructed Mehran score and preprocedural albumin < 3.5 g/dL, with the variable indicated in the first column entered as an additional covariate. The primary model is shown for reference. Odds ratios for the reconstructed Mehran score correspond to a 1-point increase. AUC values are apparent estimates with descriptive nonparametric DeLong 95% confidence intervals. All *p* values are two-sided, and no adjustment for multiple comparisons was applied to sensitivity or exploratory analyses. ^a^ The contrast component was removed from the reconstructed Mehran score, and the total contrast volume actually administered was entered separately as a continuous covariate so that contrast exposure was not counted twice. ^b^ Albumin < 3.5 g/dL was entered together with age, continuous eGFR, Mehran-defined anaemia, diabetes mellitus, and contrast volume rather than the composite Mehran score. Firth penalised estimates and the descriptive bootstrap distribution of the albumin OR among successful fits are also shown; a composite-score OR is not applicable. ^c^ Procedure duration was unavailable in four patients; this analysis was restricted to the 117 patients with complete data. Abbreviations: AUC, area under the receiver operating characteristic curve; CA-AKI, contrast-associated acute kidney injury; CI, confidence interval; CIN, contrast-induced nephropathy; CKD-EPI, Chronic Kidney Disease Epidemiology Collaboration; CRP, C-reactive protein; eGFR, estimated glomerular filtration rate; OR, odds ratio.

**Table 5:** Exploratory descriptive incidence of early CA-AKI by original Mehran category and albumin status.

Mehran Risk Category	Overall	Albumin ≥ 3.5 g/dL	Albumin < 3.5 g/dL
Low (≤5)	5/52 (9.6%; 95% CI 4.2–20.6)	1/40 (2.5%; 95% CI 0.4–12.9)	4/12 (33.3%; 95% CI 13.8–60.9)
Moderate (6–10)	16/48 (33.3%; 95% CI 21.7–47.5)	6/36 (16.7%; 95% CI 7.9–31.9)	10/12 (83.3%; 95% CI 55.2–95.3)
High (11–15)	11/21 (52.4%; 95% CI 32.4–71.7)	3/10 (30.0%; 95% CI 10.8–60.3)	8/11 (72.7%; 95% CI 43.4–90.3)

Data are presented as n/N (%; 95% CI). Confidence intervals were calculated using the Wilson method. The original Mehran risk categories were used descriptively and were not recalibrated for carotid artery stenting. No patient was classified in the very high-risk category (≥16 points). Abbreviations: CA-AKI, contrast-associated acute kidney injury; CI, confidence interval. The small subgroups and wide intervals do not allow category-specific CAS risks to be validated and do not support formal reclassification claims.

## Data Availability

A fully de-identified analytical dataset, containing no names, record numbers, or procedure dates, has been prepared and may be made available by the corresponding author subject to institutional and ethics committee approval. The analysis scripts, including the eGFR and Mehran score reconstruction and outcome classification procedures, are retained by the authors and are available upon reasonable request.

## References

[1] Davenport M.S., Perazella M.A., Yee J., Dillman J.R., Fine D., McDonald R.J., Rodby R.A., Wang C.L., Weinreb J.C. (2020). Use of Intravenous Iodinated Contrast Media in Patients with Kidney Disease: Consensus Statements from the American College of Radiology and the National Kidney Foundation. Radiology.

[2] Mehran R., Aymong E.D., Nikolsky E., Lasic Z., Iakovou I., Fahy M., Mintz G.S., Lansky A.J., Moses J.W., Stone G.W. (2004). A simple risk score for prediction of contrast-induced nephropathy after percutaneous coronary intervention: Development and initial validation. J. Am. Coll. Cardiol..

[3] Donahue M., Visconti G., Focaccio A., Selvetella L., Baldassarre M., Viviani Anselmi C., Briguori C. (2015). Acute Kidney Injury in Patients with Chronic Kidney Disease Undergoing Internal Carotid Artery Stent Implantation. JACC Cardiovasc. Interv..

[4] Spiliopoulos S., Blanc R., Gandini R., Müller-Hülsbeck S., Reith W., Moschovaki-Zeiger O. (2024). CIRSE Standards of Practice on Carotid Artery Stenting. Cardiovasc. Interv. Radiol..

[5] Pucciarelli A., Arcari A., Popusoi G., Cioppa A., Salemme L., Iacovelli F., Napolitano G., Esposito G., Tesorio T., Stabile E. (2018). Incidence and predictors of acute kidney injury in patients undergoing proximal protected carotid artery stenting. EuroIntervention.

[6] Paraskevas K.I., Mikhailidis D.P. (2017). Contrast-Induced Acute Kidney Injury in Patients Undergoing Carotid Artery Stenting: An Underestimated Issue. Angiology.

[7] Ösken A., Öz A., Keskin M., Akdeniz E., Şahan H., Şişman S.B., Çam N., Şahin S. (2021). The association between neutrophil-to-lymphocyte ratio and contrast-induced acute kidney injury in patients with carotid artery stenting. Vascular.

[8] Murat S.N., Kurtul A., Yarlioglues M. (2015). Impact of Serum Albumin Levels on Contrast-Induced Acute Kidney Injury in Patients with Acute Coronary Syndromes Treated with Percutaneous Coronary Intervention. Angiology.

[9] Wang Y., Sun W.J., Ji Z.S., Liu C.B., Wang R. (2020). Serum albumin and the risk of contrast-induced acute kidney injury after percutaneous coronary intervention. Rev. Cardiovasc. Med..

[10] Liu L., Lun Z., Wang B., Lei L., Sun G., Liu J., Guo Z., He Y., Song F., Liu B. (2021). Predictive Value of Hypoalbuminemia for Contrast-Associated Acute Kidney Injury: A Systematic Review and Meta-Analysis. Angiology.

[11] Jung J.Y., Kim A.J., Chang J.H., Lee H.H., Chung W. (2026). Inflammation and hypoalbuminemia mediate the association between CKD and contrast-associated AKI. Eur. J. Med. Res..

[12] Levey A.S., Stevens L.A., Schmid C.H., Zhang Y.L., Castro A.F., Feldman H.I., Kusek J.W., Eggers P., Van Lente F., Greene T. (2009). A new equation to estimate glomerular filtration rate. Ann. Intern Med..

[13] Inker L.A., Eneanya N.D., Coresh J., Tighiouart H., Wang D., Sang Y., Crews D.C., Doria A., Estrella M.M., Froissart M. (2021). New Creatinine- and Cystatin C-Based Equations to Estimate GFR without Race. N. Engl. J. Med..

[14] Shannon C.M., Ballew S.H., Daya N., Zhou L., Chang A.R., Sang Y., Coresh J., Selvin E., Grams M.E. (2021). Serum albumin and risks of hospitalization and death: Findings from the Atherosclerosis Risk in Communities study. J. Am. Geriatr. Soc..

[15] (2012). Kidney Disease: Improving Global Outcomes (KDIGO) Acute Kidney Injury Work Group. KDIGO Clinical Practice Guideline for Acute Kidney Injury. Kidney Int. Suppl..

[16] Riley R.D., Ensor J., Snell K.I.E., Harrell F.E., Martin G.P., Reitsma J.B., Moons K.G.M., Collins G., van Smeden M. (2020). Calculating the sample size required for developing a clinical prediction model. BMJ.

[17] Vickers A.J., Cronin A.M., Begg C.B. (2011). One statistical test is sufficient for assessing new predictive markers. BMC Med. Res. Methodol..

[18] Demler O.V., Pencina M.J., D’Agostino R.B. (2012). Misuse of DeLong test to compare AUCs for nested models. Stat. Med..

[19] Van Calster B., Nieboer D., Vergouwe Y., De Cock B., Pencina M.J., Steyerberg E.W. (2016). A calibration hierarchy for risk models was defined: From utopia to empirical data. J. Clin. Epidemiol..

[20] Vickers A.J., Elkin E.B. (2006). Decision curve analysis: A novel method for evaluating prediction models. Med. Decis. Mak..

[21] Vickers A.J., Cronin A.M., Elkin E.B., Gonen M. (2008). Extensions to decision curve analysis, a novel method for evaluating diagnostic tests, prediction models and molecular markers. BMC Med. Inform. Decis. Mak..

[22] Mehran R., Owen R., Chiarito M., Baber U., Sartori S., Cao D., Nicolas J., Pivato C.A., Nardin M., Krishnan P. (2021). A contemporary simple risk score for prediction of contrast-associated acute kidney injury after percutaneous coronary intervention: Derivation and validation from an observational registry. Lancet.

[23] Wiedermann C.J., Wiedermann W., Joannidis M. (2017). Causal relationship between hypoalbuminemia and acute kidney injury. World J. Nephrol..

